# MR and CT angiography in the diagnosis of vasculitides

**DOI:** 10.1259/bjro.20220020

**Published:** 2023-09-25

**Authors:** Alex Ghorishi, Amaris Alayon, Tarek Ghaddar, Maya Kandah, Per K Amundson

**Affiliations:** 1 Charles E. Schmidt College of Medicine, Florida Atlantic University, Boca Raton, Florida, United States; 2 School of Medicine, Indiana University, Indianapolis, United States

## Abstract

Vasculitides represent the wide-ranging series of complex inflammatory diseases that involve inflammation of blood vessel walls. These conditions are characterized according to the caliber of the predominantly involved vessels. The work-up of vasculitides often includes imaging to narrow a differential diagnosis and guide management. Findings from CT and MR angiography in conjunction with a thorough history and physical exam are of utmost importance in making an accurate diagnosis. Further, imaging can be used for follow-up, in order to monitor disease progression and response to treatment. This wide-ranging literature review serves as the primary resource for clinicians looking to diagnose and monitor the progression of rare vascular inflammatory conditions. This article provides a comprehensive summary of the main findings on imaging related to each of these vasculitides. For each of the named vasculitis conditions, a thorough overview of the diagnostic modalities and their respective findings is described. Many specific hallmarks of pathology are included in this review article.

## Introduction

Vasculitides are inflammatory processes affecting blood vessels that can be characterized by the size of the vessels involved. Historically, imaging has played a key role in the diagnosis of large vessel disease. However, as the imaging findings in small and medium vessel disease become further characterized, the clinical role of CT angiography and MR angiography are becoming more apparent in all forms of vasculitis. Large vessel vasculitides have typical vascular manifestations which will be elucidated. However, smaller vessel vasculitides may have end organ effects which may also show signs on imaging. As technology in imaging advances, greater resolutions may be achieved which will provide even greater diagnostic data. Thus, the future of vasculitis treatment will involve a close alliance between rheumatologist and radiologist for the diagnosis and management of these diseases, which can have catastrophic consequences on patients’ quality of life if not properly treated.

Inflammation of blood vessels, or vasculitis, encompasses a group of relatively rare pathologies with common clinical, laboratory, and pathophysiological features. These diseases may occur secondary to another inflammatory process, or as the primary pathology. The differences in the various vasculitides are primarily related to the size of the vessel involved and its location.^1^ The etiology of these various inflammatory processes is largely unknown, but the risk factors have been characterized. For example, Takayasu disease, a large vessel vasculitis, is more prevalent in South Asian countries. Kawasaki disease commonly affects pediatric patients less than 5 years old. Giant cell arteritis tends to afflict elderly females.^2,3^

The diagnosis and management of vasculitides can be challenging due to overlapping clinical manifestations and non-specific symptoms, like headache and abdominal pain. Thus, the role of imaging is clear, especially in large vessel vasculitides. By identifying patterns and distribution of lesions affecting blood vessels, a clinician can narrow their differential diagnosis. In vasculitis affecting small- and medium-sized vessels, imaging can identify the resultant structural and functional damage secondary to vessel involvement as markers of the underlying disease. This review will discuss each of the most common forms of vasculitis, organized by vessel size as delineated by the 2012 Chapel Hill Consensus Conference shown below in Table 1. The review will then delve into the 2018 updated European League Against Rheumatism (EULAR) recommendations, which are standardized guidelines for the management of large vessel vasculitides based on robust evidence from randomized clinical trials and cohort analyses.

**Table 1.** 2012 Chapel Hill consensus conference nomenclature of vasculitides

Large vessel vasculitis	Medium vessel vasculitis	Small vessel vasculitis	Variable vessel vasculitis	Vasculitis-associated with systemic disease	Vasculitis-associated with probable etiology
Takayasu arteritis, giant cell arteritis	Polyarteritis nodosa, Kawasaki disease	ANCA- associated vasculitis: microscopic polyangiitis, granulomatosis with polyangiitis, Eosinophilic granulomatosis with polyangiitis	Cutaneous leukocytoclastic angiitis, cutaneous arteritis, primary central nervous system vasculitis isolated aortitis	Lupus vasculitis, rheumatoid vasculitis, sarcoid vasculitis	Hepatitis C virus-associated cryoglobulinemic vasculitis, hepatitis B virus-associated vasculitis, syphilis-associated aortitis, drug-associated immune complex vasculitis, drug-associated ANCA-associated vasculitis, cancer-associated vasculitis
		Immune complex small vessel vasculitis: anti-GBM disease, cryoglobulinemic vasculitis, IgA vasculitis, hypocomplementemic urticarial vasculitis			

ANCA, antineutrophil cytoplasmic antibody; GBM, glomerular basement membrane.

### Large vessel vasculitides

#### Takayasu arteritis

Takayasu arteritis (TA) is a primary large vessel vasculitis, classically involving the aorta and its branches with possible involvement of the coronary arteries. This chronic inflammatory condition has unknown etiology but can cause significant morbidity and mortality, necessitating an early, accurate diagnosis.^4^ Imaging is the primary method of diagnosis, with conventional angiography being the historical gold-standard modality.^5^ Known to predominantly afflict young Asian females, this condition can lead to narrowing, blockage, and aneurysm formation in the aorta, brachiocephalic, common carotid, subclavian, and renal arteries. The gold-standard for diagnosis is CT angiography according to the 1994 Tokyo International Conference Classification of Takayasu Arteritis.

The more recent European League Against Rheumatism (EULAR) recommendations suggest the first-line use of MRI for the diagnosis of TA. Although their recommendation is based mostly on expert opinion and current consensus in clinical practice, MRI remains the best technique to investigate mural inflammation and luminal changes to a high degree of accuracy while avoiding radiation exposure.^6^ Since the disease most commonly afflicts those between 40 and 50 years of age, the avoidance of exposure to ionizing radiation should be prioritized.^7^

Further, MRA demonstrates remarkable diagnostic accuracy for TA. Yamada et al examined the efficacy of MRA for establishing the diagnosis of TA in 30 consecutive patients with suspected TA by comparing MRA results against conventional angiography. 20 of the 30 patients were diagnosed with TA. Their ages ranged from 23 to 63 years (mean of 42 years of age). The researchers achieved an estimated sensitivity and specificity of 100% using a 1.5 T superconducting system with a 25 mT/m maximum gradient capability (Magnetom Vision; Siemens, Erlangen, Germany) and a body phased-array coil. Scout images consisted of conventional sagittal, coronal, and axial sections of the thorax and upper abdomen. A three-dimensional (3D) fast low-angle shot (FLASH) sequence was used to obtain breath-hold contrast-enhanced 3D MR angiograms with a 25 s acquisition time using a TR/TE of 4.4/1.3 ms, 30-degree flip angle, pixel size of 1.6 × 0.8 mm and effective section thickness of 1.5 mm after interpolation. Gadodiamide contrast was administered via bolus at 0.1 mmol/kg (Omniscan; Daiichi Seiyaku, Tokyo, Japan) by manual injection at approximately 2 ml/s followed by a 20 ml injection of normal saline through an 18-gauge intravenous catheter. The imaging sequence was initiated 5 s after the start of contrast injection. Pre-contrast imaging was used as a subtraction mask. Subsequently, maximum intensity projection (MIP) algorithm was used to construct the MR angiograms in various view directions.^8^

**Figure 1.**
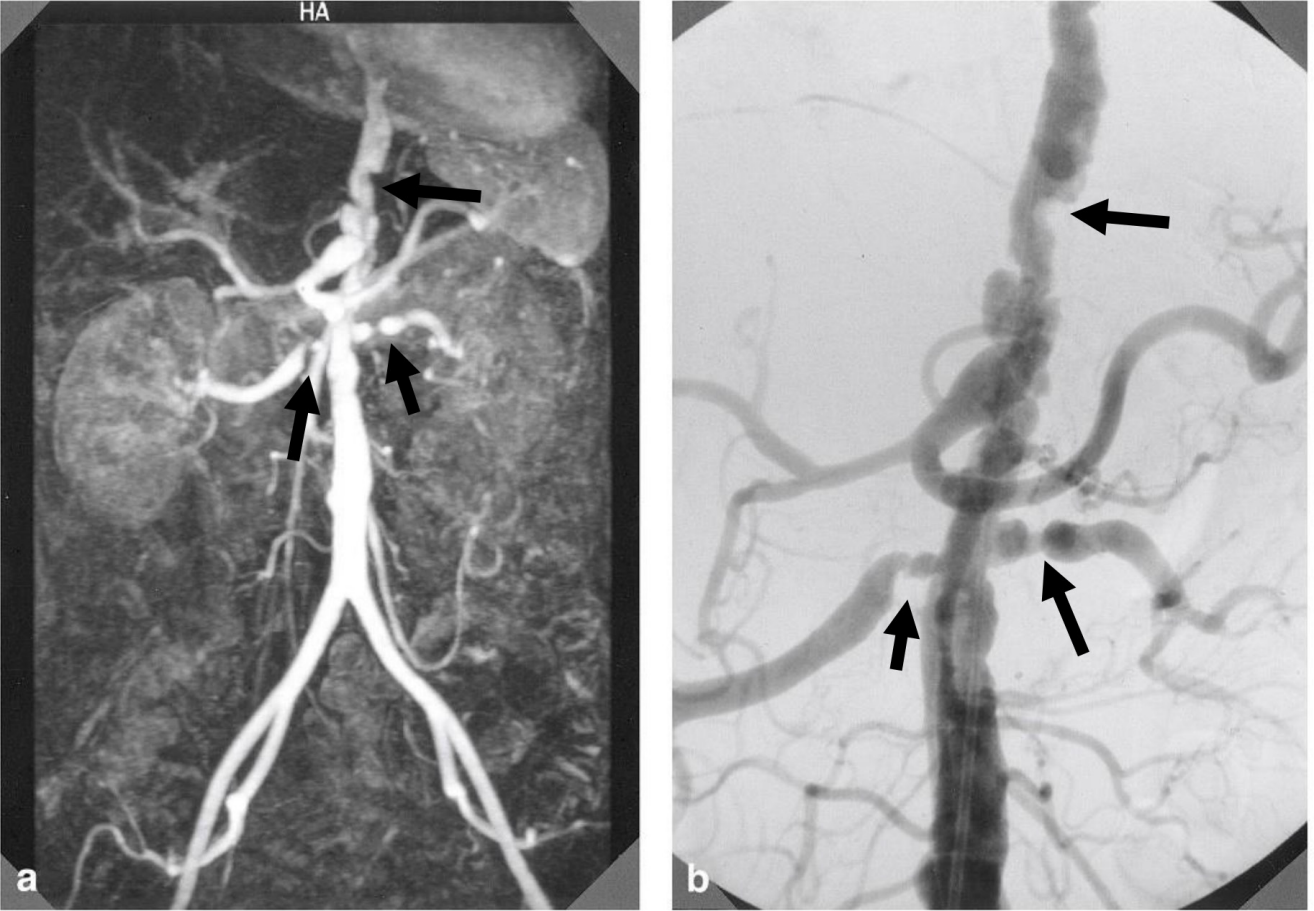
(**a**) MIP of MR angiogram (TR/TE 4.4/1.4 ms; anteroposterior view) obtained from a 55-year-old female diagnosed with TA demonstrating a series of irregular stenoses involving the abdominal aorta and bilateral renal arteries (arrows). (**b**) Corresponding vascular irregularities in the same patient as seen on abdominal aortography (arrows). MIP, maximum intensity projection; TA, Takayasu arteritis; TE, echo time; TR, repetition time.

The MR angiograms obtained by Yamada et al were assessed for vascular lesions, *i.e.* stenosis, occlusion, dilatation, and aneurysms, in the ascending aorta, aortic arch, descending aorta, abdominal aorta, brachiocephalic trunk, bilateral subclavian arteries, bilateral common carotid arteries, and bilateral vertebral arteries. The results from MRA were compared with diagnostic evidence for TA from conventional angiography—including the presence of occlusive lesions or dilatations in the aorta, its major branches or both. Typical pathologic features of TA are depicted in Figure 1a-b.^8^

MRA correctly diagnosed all twenty patients with TA, resulting in an estimated sensitivity of 100%. Further, MRA correctly identified all 10 patients without TA as not having the diagnosis, yielding 100% specificity. Further analysis of the Yamada et al study shows that the most common manifestations of TA are stenoses in the descending thoracic aorta and dilatations in the ascending aorta.^8^

However, limitations to MRA assessment of the great vasculature in patients with suspected TA do exist. Specifically, MRA tends to exaggerate stenoses compared to conventional angiography, to the point of suggesting false occlusions. Hypotheses for the cause of these MRA misrepresentations include spin dephasing caused by turbulent blood flow in narrowed vessels *vs* suboptimal spatial resolution.^9,10^

Although vascular stenosis secondary to TA can be misattributed to manifestations of atherosclerotic cardiovascular disease, the identification of disease affecting the pulmonary arterial system is specific to TA. Thus, the effectiveness of MRA for the diagnosis of TA can be augmented by radiologist’s careful assessment of luminal variabilities in the pulmonary vasculature.^11–13^ Further, MRA holds promise in monitoring the progression of TA. EULAR’s recommendations 10 and 11 corroborates the use of imaging for investigating acute flares and monitoring long-term structural damage in the setting of established large vessel vasculitis.^6^ Although these recommendations are based on expert opinion alone, imaging may provide enough evidence to alter treatment plans, especially when clinical and laboratory findings are inconclusive. Image-based monitoring and follow-up of patients with TA should be decided on an individual basis, with no universal algorithm for determining indications of various studies. Clinical judgment based on the presence of symptoms associated with stenosis and/or aneurysms should be used when deciding to pursue follow-up imaging. Further, location of suspected vascular involvement can drive decision-making in choosing the appropriate imaging modality*—i.e.* ultrasound in case of axillary/subclavian arteries versus MRA/CTA in the work-up of patients with suspected aortic involvement.

**Figure 2.**
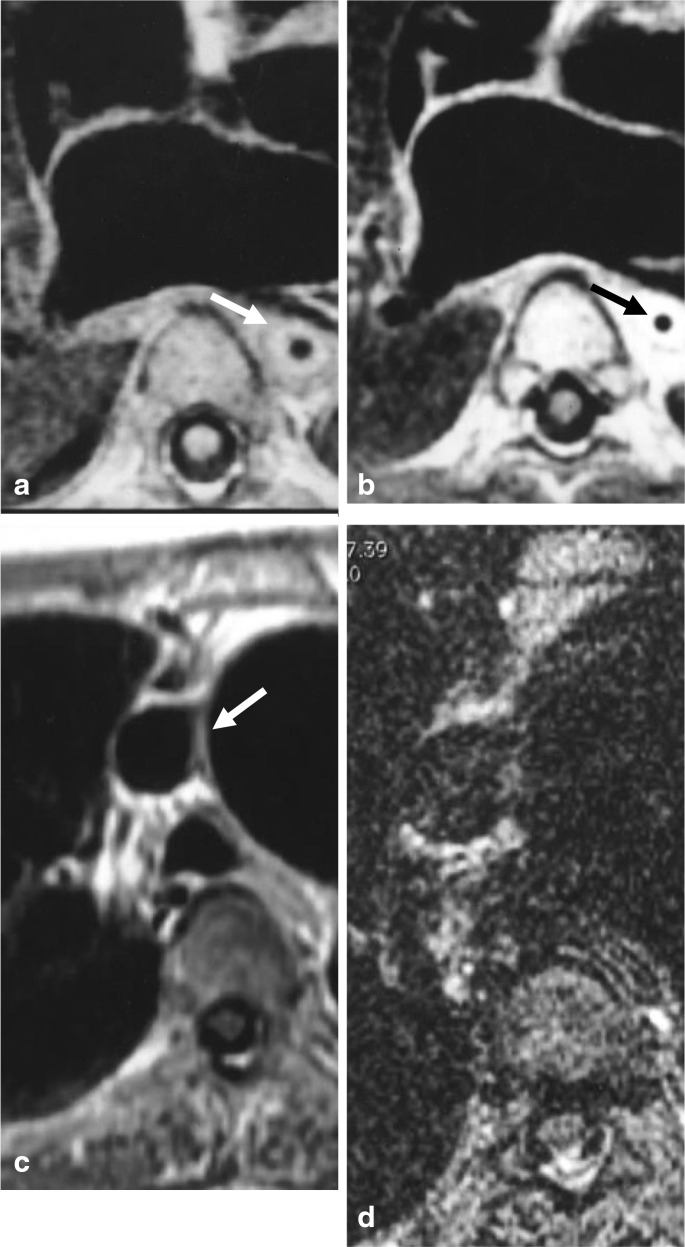
(**a**) Unenhanced *T*_1_ weighted MRI depicting marked vascular thickening of the descending thoracic aorta (arrow) in a 4-year-old girl with active TA. (**b**) Early contrast-enhanced *T*_1_ weighted MRI demonstrating significantly increased signal intensity of the aortic wall (arrow) and the surrounding tissue compared to myocardial enhancement, consistent with inflammation due to TA in the same 4-year-old TA patient. (**c**) Contrast-enhanced *T*_1_ weighted MRI in 42-year-old female with inactive TA after 5 months of medical treatment demonstrating chronic vascular changes associated with TA, *i.e.* thickened aortic wall and dilated ascending aorta (arrow), without signs of active aortitis (minimal aorticwall enhancement).(**d**) *T*_2_ weighted contrast-enhanced MRI of same 42-year-old female demonstrating lack of findings associated with mural edema. Reproduced with permission of the American Roentgen Ray Society from Takayasu’s arteritis: assessment of disease activity with contrast-enhanced MR imaging, Choe YH, Han BK, Koh EM, Kim DK, Do YS, Lee WR, *American Journal of Roentgenology*, 175, 2, 505–11 Copyright^©^ 2000 American Roentgen Ray Society. TA, Takayasu arteritis.

Several studies have shown the applicability of contrast enhanced MRI to stratifying active *vs* inactive TA. In one study involving 26 patients with TA and 16 healthy subjects, contrast-enhanced MRI assessment of disease activity was consistent with clinical findings in 23 patients (88.5%). Further, determination of active disease by imaging was concordant with laboratory findings in most patients. Elevated ESR in 24 patients (92.3%) and elevated C-reactive protein level in 22 patients (84.6%) were found in the 26 patients with active TA as determined by contrast-enhanced MRI.^14^ Comparison of imaging findings in patients with active TA *vs* inactive TA are clearly depicted in Figure 2a–d (Reproduced with permission of the American Roentgen Ray Society from Takayasu’s arteritis: assessment of disease activity with contrast-enhanced MR imaging, Choe YH, Han BK, Koh EM, Kim DK, Do YS, Lee WR, *American Journal of Roentgenology*, 175, 2, 505–11 Copyright^©^ 2000 American Roentgen Ray Society).^14^

**Figure 3.**
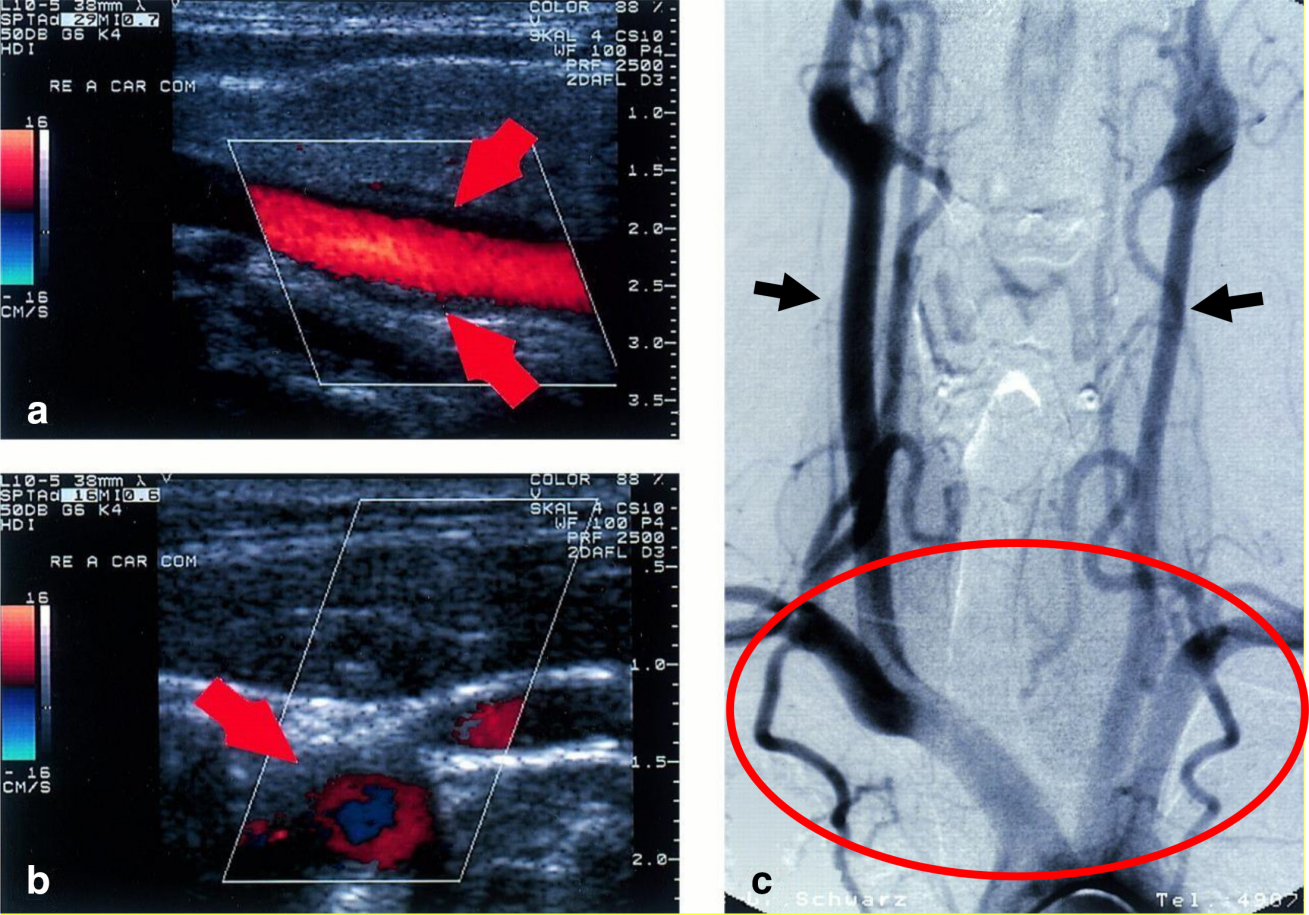
(**a**) Longitudinal plane view of characteristic ultrasound finding in early TA. (b). Transverse plane view of the same finding. (c). Angiographic confirmation of the diagnosis of TA, revealing 40% stenosis of the right common carotid artery with accompanying post-stenotic dilations well visualized in the right common carotid artery, left proximal common carotid artery, proximal right vertebral artery, and proximal right subclavian artery (circle). Note difference in thickness of right and left common carotid arteries (arrows). Reproduced from Schmidt, W.A,; Nerenheim, A., Diagnosis of early Takayasu arteritis with sonography, Rheumatology, 2002, 41, five by permission of Oxford University Press. TA, Takayasu arteritis.

In cases of suspected TA where MRI is unavailable, EULAR recommends the use of PET, CT, and/or ultrasound as alternative imaging modalities for establishing the diagnosis of TA based on expert opinion alone rather than concrete evidence-based analysis.^6^ However, it should be noted that ultrasound holds limited value for assessment of the thoracic aorta but can be especially useful in patients presenting with upper or lower limb claudication as the involved vessels are located more superficially. Endoscopic ultrasound can be a suitable technique for assessing aortic involvement in TA.

The manifestations of TA on ultrasound have been well characterized. For example, Schmidt et al describe the characteristic appearance of a homogenous, midechoic, circumferential thickening of the common carotid artery, as depicted in Figure 3.^15^

**Figure 4.**
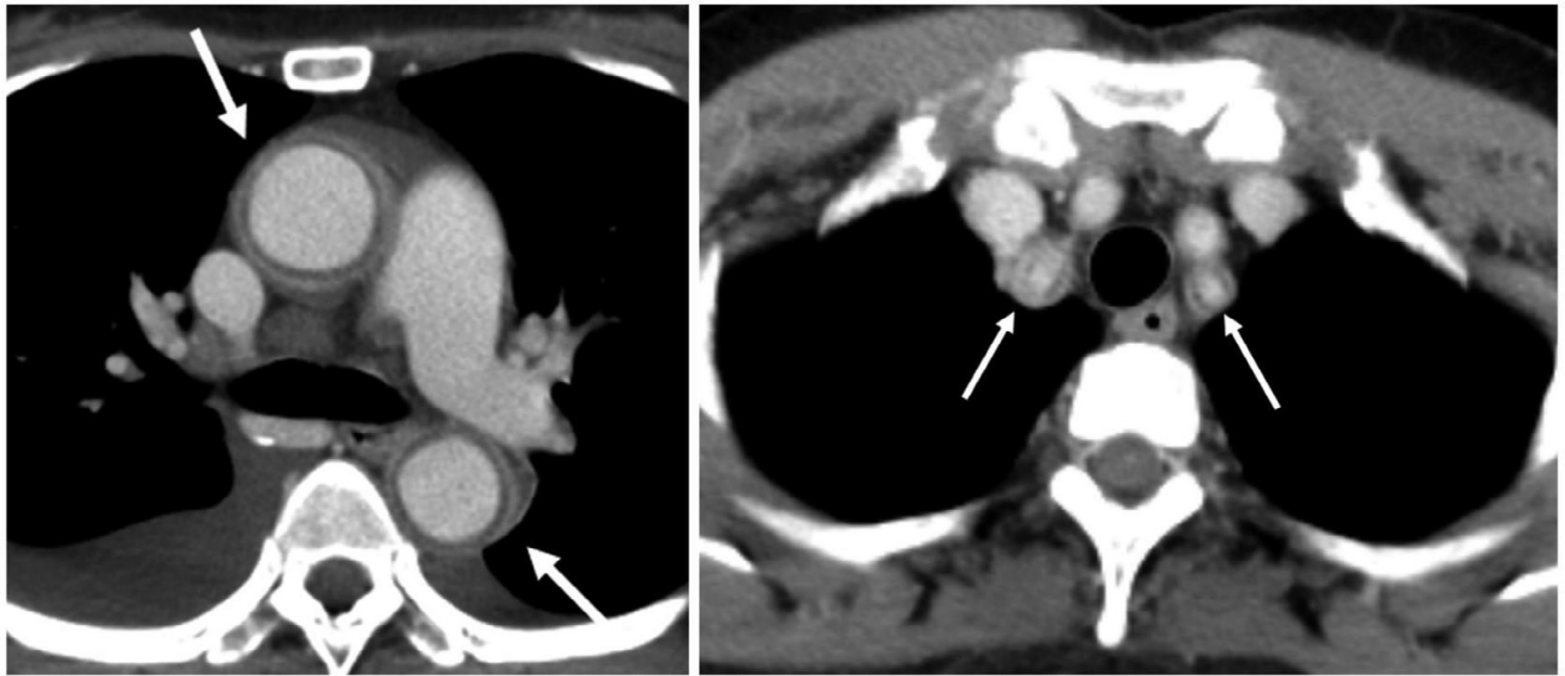
Early phase axial CT scan demonstrating typical concentric wall thickening with double rings, note that the inner ring is hypodense compared to the relatively hyperdense outer ring. The image on the left shows involvement of the ascending and descending aorta while the image on the right demonstrates involvement of the bilateral proximal subclavian arteries.

CT angiography in TA typically demonstrates concentric mural thickening in the diseased arteries, with potential calcifications in these thickened walls.^16^ Contrast-enhanced CTA may show a double ring enhancement pattern, as seen in Figure 4.^17^ An inside ring remains poorly enhanced while a larger, circumscribing ring becomes far more dramatically enhanced.^18^ Some authors suggest the inside ring corresponds to an edematous intimal layer while the outside ring represents ongoing acute inflammation in the medial and adventitial portions of the vessel wall.^19^ The result of this mural thickening is concentric narrowing of the vessel lumen.

**Figure 5.**
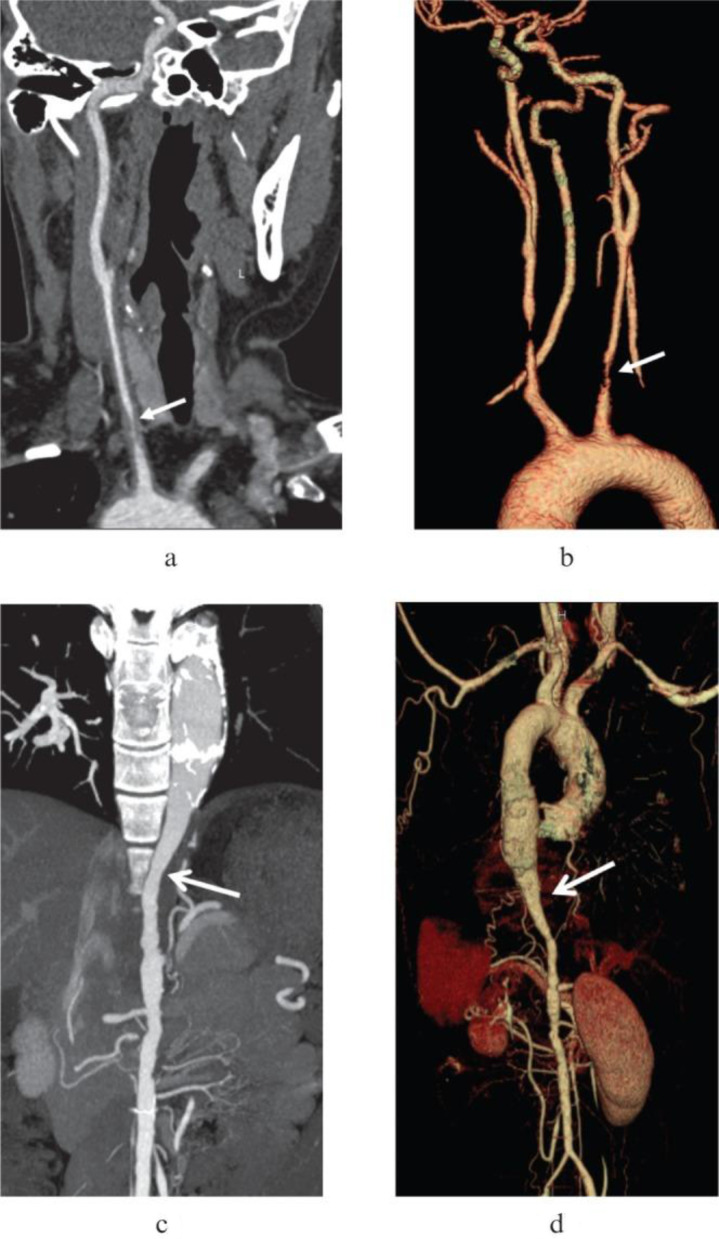
(**a**) Coronal view of severely stenotic lesion in the left common carotid artery. (b). 3D reconstruction of the same finding. (c). Maximum intensity projection of dilation of proximal descending thoracic aorta with corresponding stenotic distal segment, producing characteristic “rat-tail” sign in TA. (d). Volume-rendered 3D reconstruction of the same finding. 3D, three-dimensional; TA, Takayasu arteritis.

Over 60% of patients with TA exhibit luminal stenosis of the descending aorta, dubbed the “rat-tail sign”, depicted in Figure 5a-d.^20^ Although aneurysms are most found in the aorta with TA, they remain a rare complication. In one study, only 4% of patients at a large Chinese hospital suffered from aortic aneurysm related to their underlying TA diagnosis.^21^

**Figure 6.**
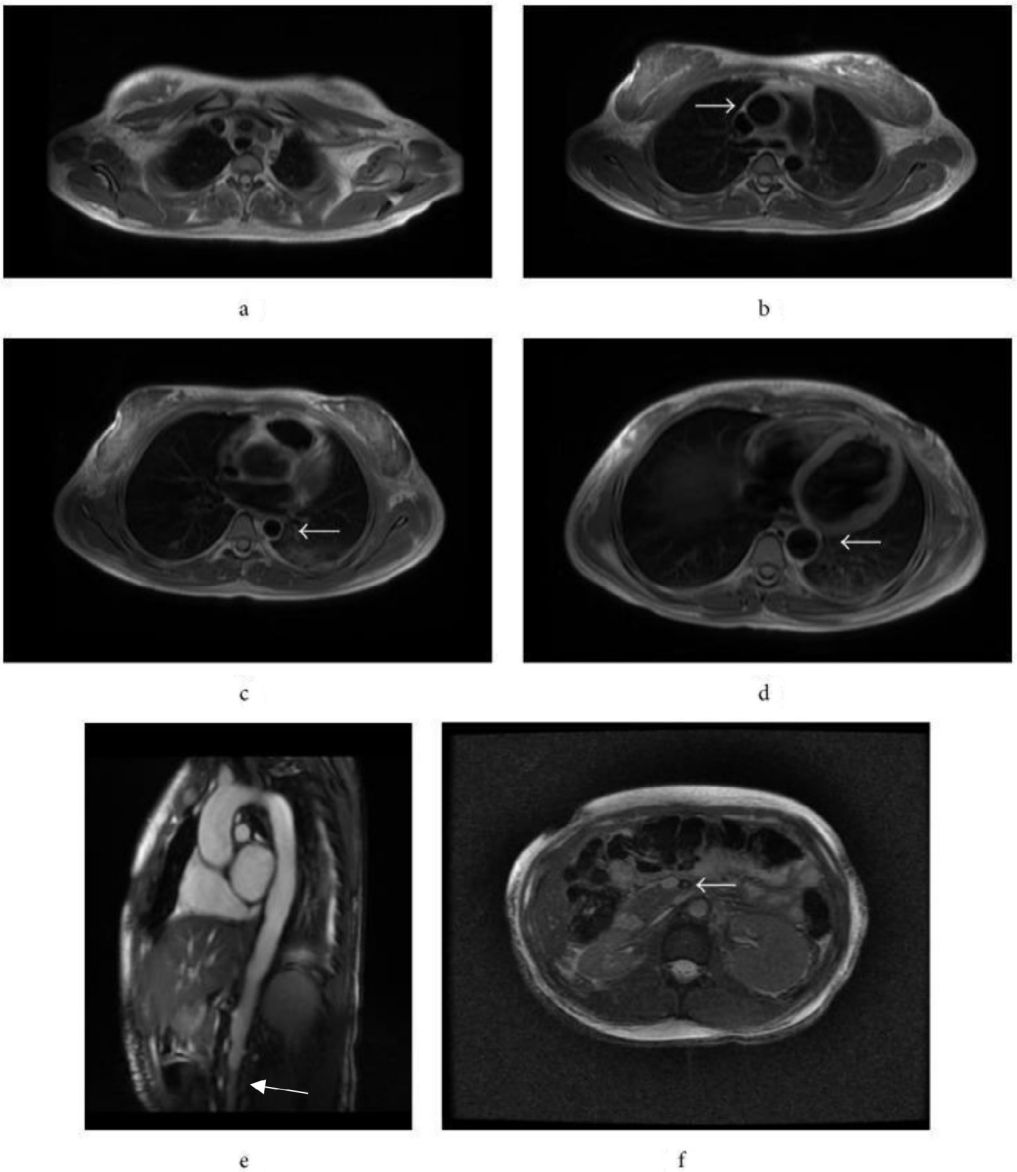
(**a**) Mural thickening of the brachiocephalic, left common carotid, and left subclavian arteries as seen on MRI/MRA of the chest, abdomen, and pelvis. (**b**). Similar study demonstrating mural thickening of the ascending aorta, measuring 3.4 cm in diameter. (c). Focal narrowing of descending aorta, measuring 2.2 cm. (d). Dilation of descending aorta distal to the proximal stenosis seen in 6c. (e). Irregular abdominal aorta vascular wall on sagittal cine-FIESTA sequence. (f). Thickening and mural narrowing of the superior mesenteric artery on axial T2 FIEST fat-saturated sequence. MRA, MR angiography.

**Figure 7.**
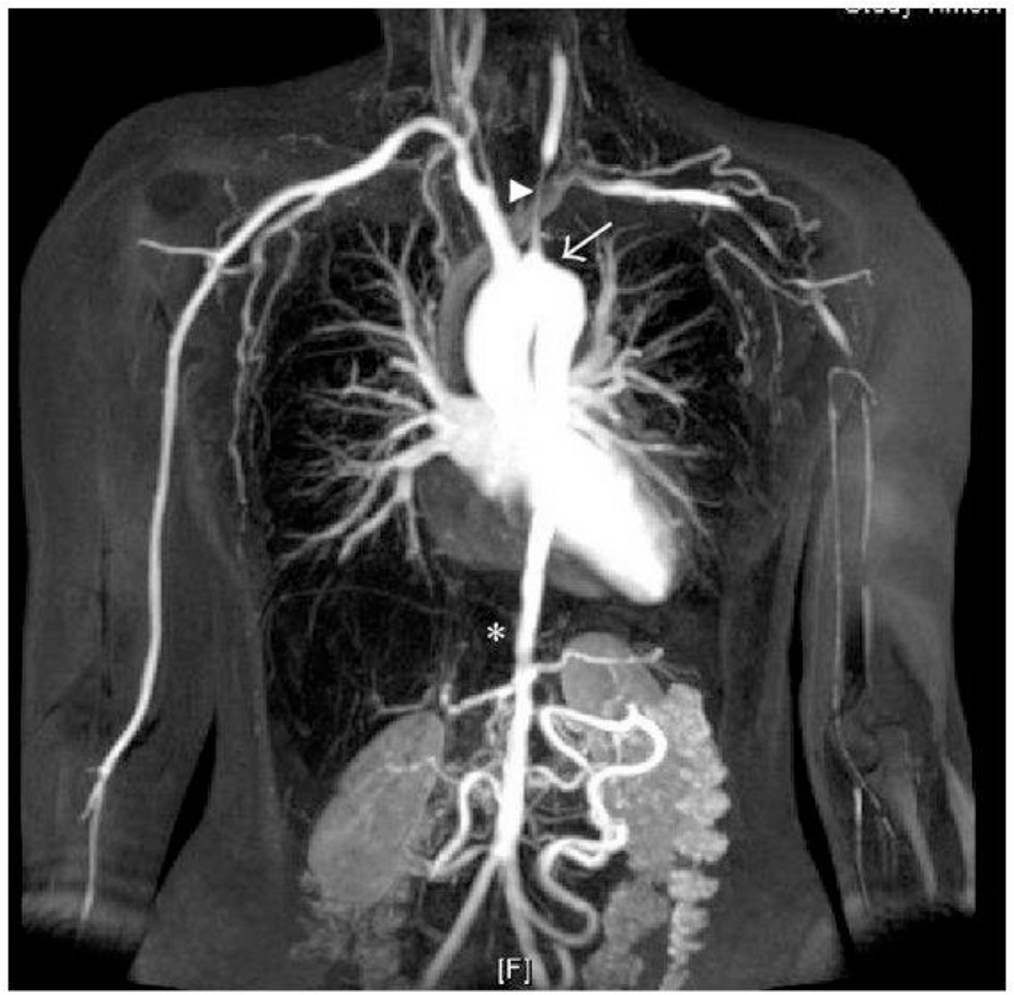
Contrast-enhanced MRA of a young female with TA, positive for an occluded left subclavian artery (solid arrow), stenosis of the left common carotid artery (arrowhead), and irregularly narrowed thoracic and abdominal aorta (star). TA, Takayasu arteritis.

MR angiography represents the most significant imaging modality in the diagnosis of TA, particularly in early disease and in patient populations more susceptible to the harmful effects of ionizing radiation like children. Mural edema, wall thickening, and wall enhancement are better characterized on MRI and MRA, seen in Figure 6.^22^ Further MRA depicted in Figure 7 clearly resolves the large vessel abnormalities typical of TA.^23^ ^96^ Thus, a diagnosis may be established before the development of overt clinical symptoms from vessel stenosis and occlusion.^24^ Although CTA does demonstrate mural thickening, there is less contrast between the vessel wall and vessel lumen due to the presence of intraluminal contrast. However, through black-blood MR sequence, better visualization of the mural thickening and enhancement is achieved. Ultimately, there is widely accepted consensus that recommends against the use of conventional angiography for the diagnosis of any large vessel vasculitis.^6^

#### Giant cell arteritis

Giant cell arteritis (GCA), also known as temporal arteritis, is the most common form of vasculitis in adults over 50 years old, affecting medium- to large-sized arteries. Notably, this condition can lead to irreversible blindness from ischemic optic neuropathy if unrecognized. Numerous arteries may be involved including the aorta, subclavian, iliac, ophthalmic, occipital, and vertebral, but the temporal artery is the most implicated. Although the gold-standard for diagnosis is temporal artery biopsy, up to 44% of GCA patients will have a normal biopsy.^25^ An early, accurate diagnosis is crucial for treatment decisions. Treatment cessation may lead to blindness in undiagnosed patients, while unnecessary treatment may increase the risk of glucocorticoid side effects.

MR modalities have demonstrated utility in the diagnosis of GCA, albeit at a higher cost than CTA and ultrasound. The increased level of detail offered by MRI allows sharp visualization of the superficial temporal artery, offering the clinician a detailed view of the vessel lumen and wall. Thus, more minute manifestations of inflammatory vasculitis changes may be detected with this modality. Sensitivities and specificities approaching 100% have been reported in small-volume studies.^26^ A subsequent prospective three-university medical center trial demonstrated high diagnostic accuracy for MRI in GCA patients, with a sensitivity of 78.4% and specificity of 90.4%. However, both MRI/MRA and CTA are limited in their diagnostic capabilities after initiation of glucocorticoid therapy.^27^

The European League Against Rheumatism (EULAR) has developed evidence-based recommendations for the use of imaging modalities in primary large vessel vasculitis (LVV), specifically GCA and TA. The 20-member task force consisting of rheumatologists, radiologists, nuclear medicine specialists, patient representatives, an internist, and a methodologist formulated the following recommendations based on a hybrid of evidence and expert opinion. In GCA, the task force recognized the validity of temporal artery biopsy as the gold-standard diagnostic test for GCA. However, the EULAR guidelines recommend imaging as a first-line diagnostic test because of low invasiveness in settings where imaging is easily accessible.^6^ The task force supports the first-line use of temporal and/or maxillary artery ultrasound in all patients with suspected GCA, as it is cost-effective compared with biopsy when considering the costs of the tests and the consequences of an incorrect diagnosis, *i.e.* side effects of unnecessary steroid use or inadvertent vision loss due to delays in prompt treatment.^25^ However, it must be noted that vascular ultrasound is strongly operator- dependent. In settings with little expertise in imaging for GCA, temporal artery biopsy may remain the preferred diagnostic technique.

**Figure 8.**
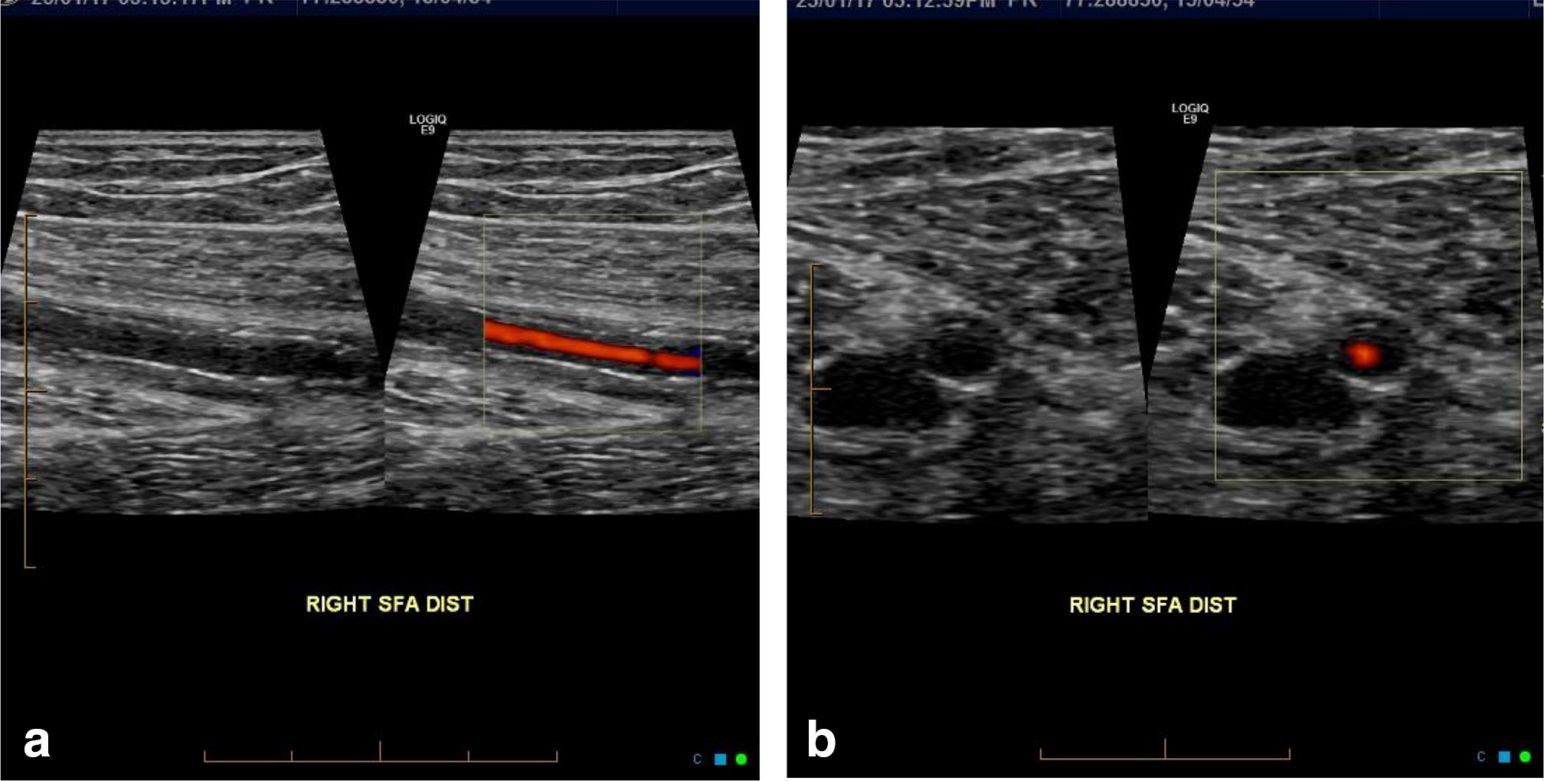
(**a**) Longitudinal view of halo sign. Hollow white arrows demarcating accumulation of edema in the vascular wall associated with GCA. (**b**) Transverse view of similarly involved temporal artery frontal branch. GCA, giant cell arteritis.

A positive vascular ultrasound for GCA has four pathological hallmarks which include wall thickening with halo sign, non-compressible arteries, stenosis, and vessel occlusion. GCA primarily involves edematous infiltration of the media which may extend to the intima and adventitia. Figure 8a and b demonstrate hypoechoic material around the artery, referred to as the *halo sign*, lumen suggests GCA-associated edematous wall thickening.^28^

**Figure 9.**
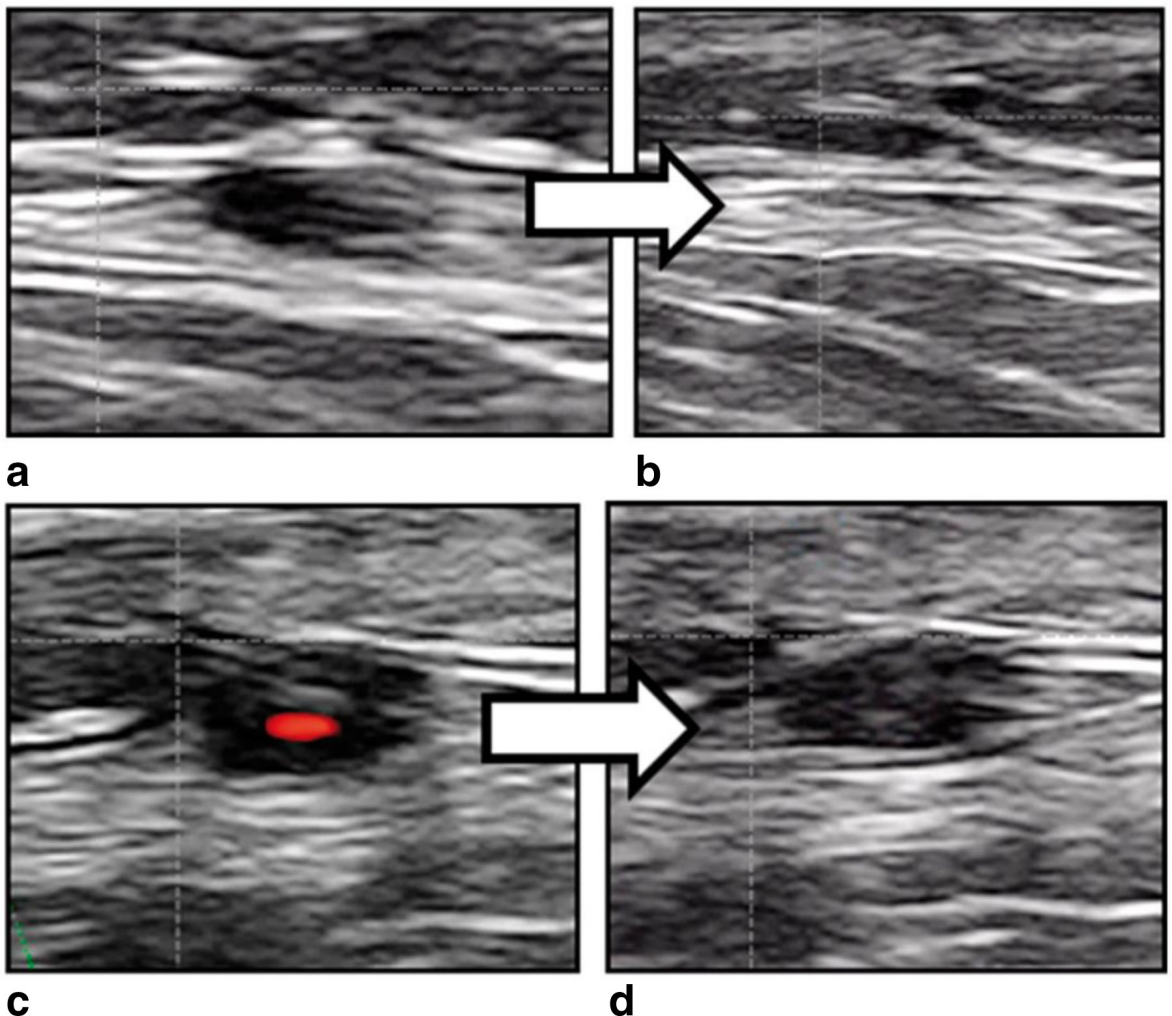
(**a**) Normal temporal artery prior to compression. (b). Normal temporal artery collapsing under pressure. (c). Inflamed temporal artery prior to compression. (d). Inflamed temporal artery resistant to compression, suggestive of the diagnosis of GCA.Reproduced from Schmidt, AW, Ultrasound in the Diagnosis and Management of Giant Cell Arteritis, Rheumatology, 2018, 57, 2, by permission of Oxford University Press. GCA, giant cell arteritis

Non-compressible temporal arteries are another finding on ultrasound consistent with the diagnosis of GCA. Color doppler ultrasonography allows easy detection of the presence of blood flow during compression of the temporal artery with the ultrasound probe. Since the temporal artery is quite superficial, occlusion of blood flow is easily achievable with little application of pressure through the ultrasound probe. However, inflammatory tissue in the vessel wall is characterized by its decreased compressibility, giving rise to the compression sign. Figure 9a–d compares the appearance of a positive *vs* negative compression sign in temporal artery ultrasound (Schmidt, AW, Ultrasound in the Diagnosis and Management of Giant Cell Arteritis, Rheumatology, 2018, 57, 2, by permission of Oxford University Press).^29^ The compression sign is a variant of the halo sign as it signifies the presence of incompressible hypoechoic vascular wall swelling, with sensitivities of 77–79% and specificity of 100% for the diagnosis of GCA.^30,31^

Patients with clinical signs and symptoms concerning for GCA and positive ultrasound results can be diagnosed with GCA without additional testing, whereas those with no symptoms and negative ultrasound are unlikely to have GCA. In any other clinical scenario, further testing is necessary. For example, equivocal ultrasound results may be followed up by high resolution MRI of cranial arteries for the detection of mural inflammation. Although MRI has similar diagnostic value to ultrasound, the restricted availability, higher cost, and potential complications of gadolinium contrasts are disadvantages to MRI testing.^32^

**Figure 10.**
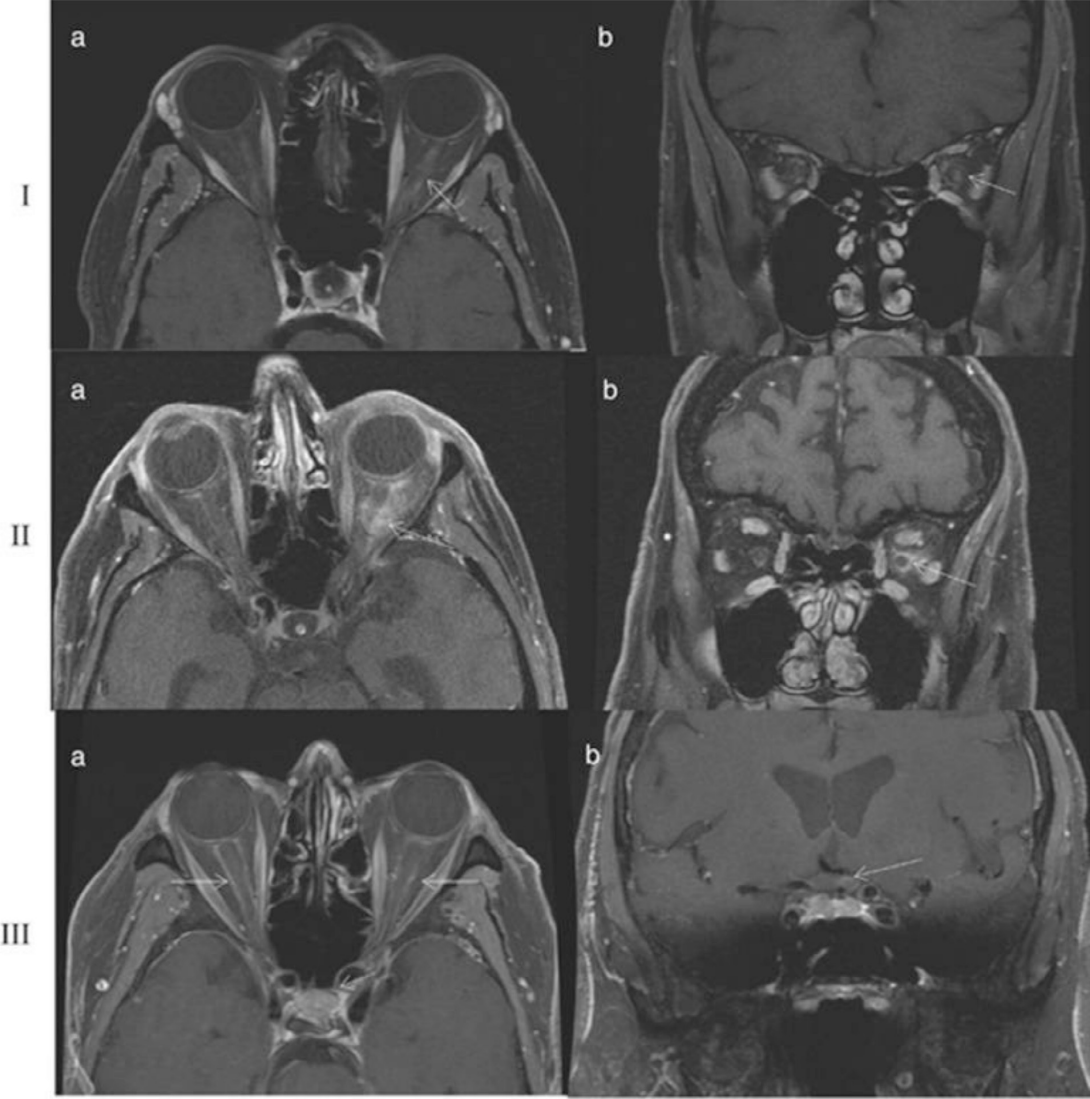
(Ia) Contrast-enhanced axial post-gadolinium *T*_1_ weighted orbital MRI with fat suppression demonstrating left optic nerve enhancement. (Ib) Coronal view of same case. (IIa) Contrast-enhanced axial post-gadolinium *T*_1_ weighted orbital MRI with fat suppression demonstrating enhancement of the left optic nerve sheath. (IIb) Coronal view of same case. (IIIa) Bilateral enhanced, enlarged perineural sheath involving both optic nerves. (IIIb) Coronal view of same case demonstrating enhancement of the optic chiasm (rare). Magnetic resonance imaging findings in giant cell arteritis, N M D’Souza et al, Eye, Springer Nature, 2016, reproduced with permission from SNCSC.

Cranial MRI technique requires a 1.5 T, or preferably 3.0 T MRI scanner with minimum 80 channel head-coil. Studies should include *T*_1_ weighted spin echo with gadolinium-contrast enhancement, fat suppression, and high in plane resolution (<<1 mm^2^). Transversal slices should be angulated parallel to the skull base.^6^ Typical MRI findings in patients with active GCA include dural and perineural sheath enlargement, sheath and nerve enhancement, and vascular changes to extracranial and intracranial vessels. Figure 10 demonstrates classical MRI findings in three different cases of GCA (Magnetic resonance imaging findings in giant cell arteritis, N M D’Souza et al, Eye, Springer Nature, 2016, reproduced with permission from SNCSC).^33^

Lastly, some authors suggest MRI may prove useful for distinguishing between arteritic-anterior ischemic optic neuropathy (A-AION) secondary to GCA versus non-arteritic anterior ischemic optic neuropathy (AION). Non-arteritic AION typically lacks gadolinium enhancement, unlike A-AION. In conclusion, the four main MRI findings in GCA—orbital enhancement, optic nerve parenchymal enhancement, perineural sheath enhancement, and optic chiasmal enhancement—remain non-specific and must be interpreted in the context of the patient’s clinical presentation. However, these findings are sufficiently suggestive of GCA to not warrant further delay of glucocorticoid treatment due to concerns of potential vision loss.^33^

**Figure 11.**
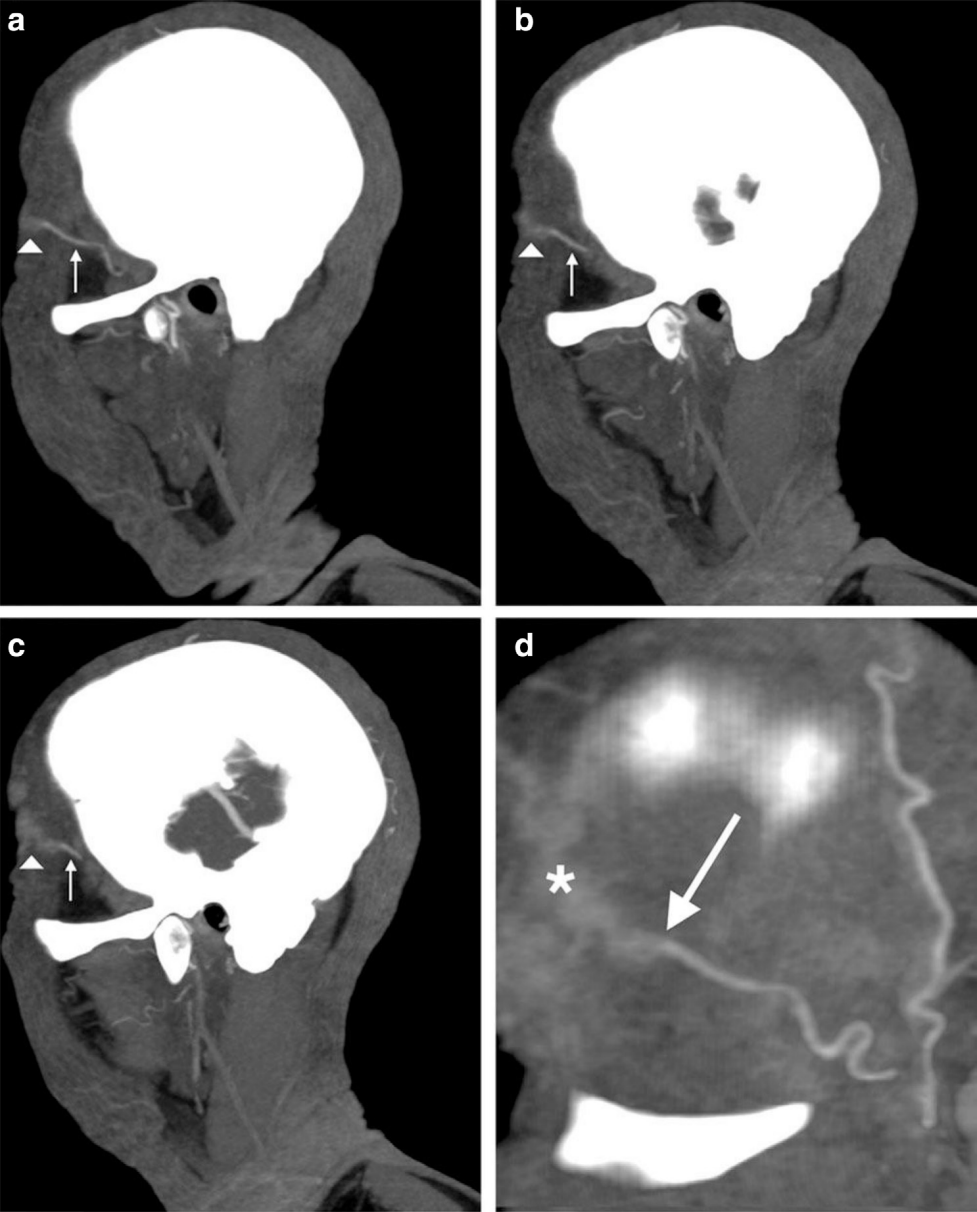
Cigar smoke sign on sagittal maximum intensity projection CTA images from a 71- year-old male with GCA. (**a–c**) Left superficial temporal artery is depicted with a normal proximal segment, with less well-defined distal segment, represented by the arrow and arrowhead, respectively. (**d**) Magnified view of blurred soft-tissue enhancement in the distal left superficial temporal artery. Reproduced from Conway, R., Smyth, AE, Kavanagh, RG, et al, Diagnostic Utility of Computed Tomographic Angiography in Giant Cell Arteritis, Stroke, 49, 9, by permission of Wolters Kluwer Health, Inc. CTA, CT angiography; GCA, giant cell arteritis

Despite EULAR task force recommendations against the use of CT or PET for the assessment of cranial artery inflammation, CTA has shown promise in revolutionizing the diagnosis and management of GCA. A 2018 case–control study demonstrated a sensitivity and specificity of 71.4 and 85.7%, respectively.^26^ Characteristic findings on CTA in GCA cases were contiguous areas of normal vessel followed by abnormal regions with blurred vessel margins, akin to smoke arising from the end of a cigar, see Figure 11 (reproduced from Conway, R., Smyth, AE, Kavanagh, RG, et al, Diagnostic Utility of Computed Tomographic Angiography in Giant Cell Arteritis, Stroke, 49, 9, by permission of Wolters Kluwer Health, Inc.).^26^ Further, CT and PET/CT are especially useful for assessment of GCA in patients with involvement of the great vessels which cannot be visualized with external ultrasound techniques. EULAR’s recommendation six corroborates this conclusion, especially in the setting of aortitis.

**Figure 12.**
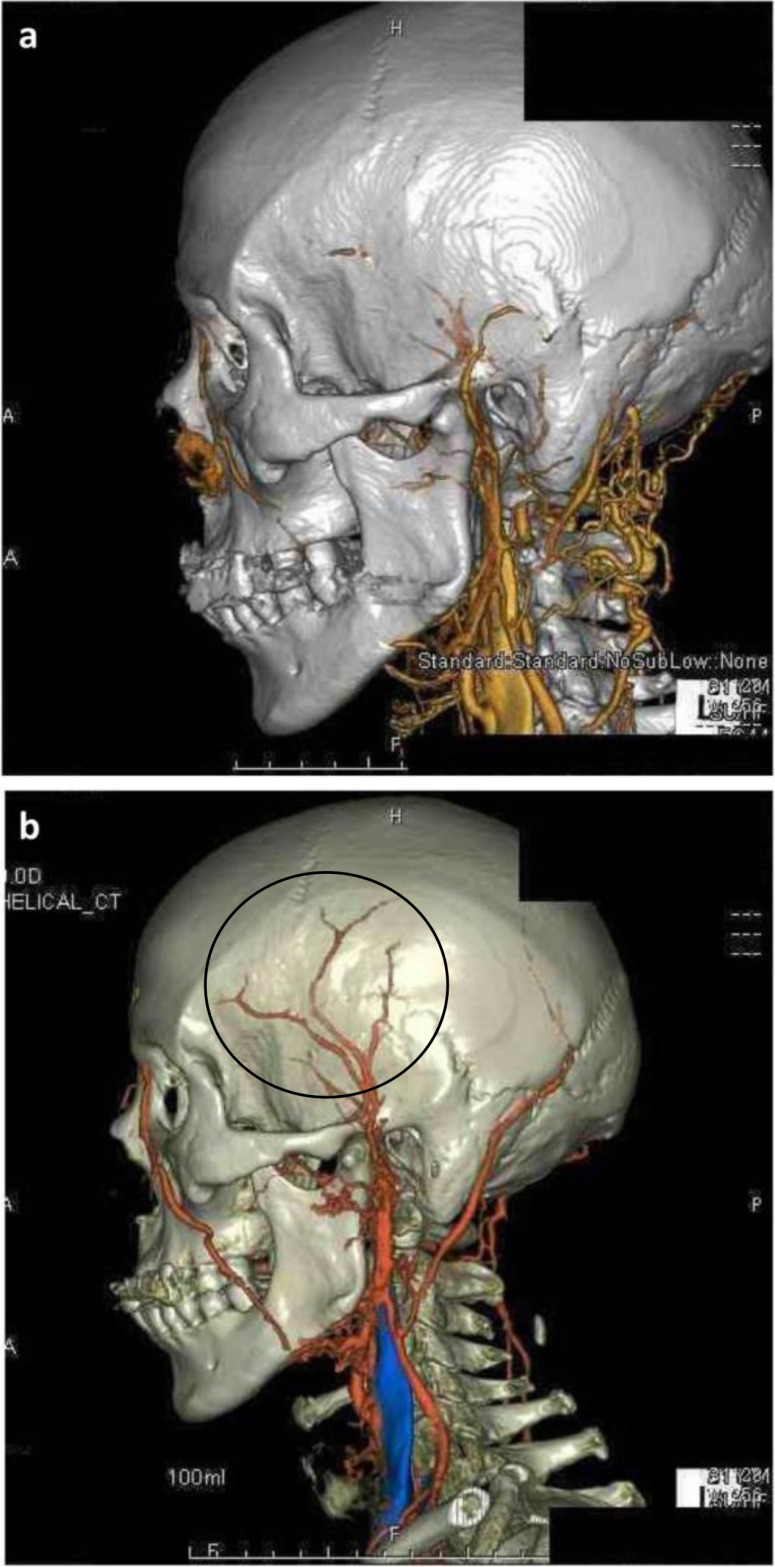
(**a**) 3D CTA prior to therapeutic immunosuppressive therapy for GCA. (b). 3D CTA after therapeutic immunosuppressive therapy with glucocorticoids demonstrating opening of the temporal artery (circle). 3D, three-dimensional; CTA, CT angiography; GCA, giant cell arteritis.

Improved reconstruction techniques for building 3D CTAs have increased their diagnostic usefulness to the point of potentially supplanting the need for temporal artery biopsy altogether. Routine temporal artery biopsies are invasive and imperfect, with occasional false-negative results. Furthermore, only few institutions have the capabilities to biopsy the temporal artery.^34,35^ 3D CTA can characterize the stenosis and blockage of blood flow in the temporal artery to great detail. Radiographic evidence in conjunction with clinical findings such as fever and jaw claudication in conjunction with elevated laboratory inflammatory markers are sufficient for establishing the diagnosis of GCA.^35^ Figure 12 demonstrates 3D CTA of a GCA patient before and after immunosuppressive therapy.^35^ Note the resolution of temporal artery stenosis and blockage after 4 weeks of prednisolone 40 mg by mouth daily. CTA was bolus tracked from the aortic arch by injecting 100 ml of non-ionic, water-soluble contrast injected at 4 ml s^−1^ (ECG triggering is another suitable option). Recommended tube voltage of 120 kV with collimation of 0.6 mm used. The study generated 8-mm-thick maximum intensity projection reconstructions with 2-mm reconstruction increments in axial, sagittal, and coronal planes with axial 0.75 mm slices.

Fluorine 18-fludeoxyglucose (FDG) positron emission tomography (PET) combined with CT (PET/CT) may be used to determine whether there is active involvement of GCA. Inflammatory processes tend to uptake FDG due to the increased glucose consumption associated with increased metabolic activity in the vessel walls affected by arteritis. Further hybrid imaging with FDG PET/MRI can be used to aid diagnosis and follow-up for improvement during treatment of GCA and other large vessel vasculitides. Increased morphological detail afforded with MRI can increase detection sensitivity in the setting of multiple metabolically active vascular segments.^36^ Supplementary figure 13 demonstrates elevated radiotracer uptake as indicated by the arrows in the walls of the aortic arch, indicating active inflammation associated with GCA. For adequate study quality, blood glucose levels below 126 mg dl^−1^ are preferred (with levels below 180 mg dl^−1^ acceptable). Further, an interval between FDG infusion and image acquisition of at least 60–90 min is necessary to allow sufficient time for adequate radiotracer uptake. The studies should be acquired with the patient supine, with arms positioned by the patient’s sides. The patient should be scanned from the top of the head to the knees to allow for adequate comparison with background uptake.^37^

Supplementary Figure 13.

Further studies must be performed to better characterize the performance of CTA and MRA in the diagnosis of GCA. However, due to the relatively low specificity of relying on temporal artery biopsy alone, imaging holds great promise in the future management of these patients. Although ultrasound has well-characterized uses for the diagnosis and management of patients with potential GCA, other modalities such as MRA, and even 3D CTA, hold great promise when ultrasound is unavailable or equivocal. The need for invasive biopsy procedures could be eliminated with the development of imaging-based diagnostics for GCA.

#### Medium vessel vasculitides

“Medium-vessel” refers to visceral arteries and veins as well as their branches. Supplementary figure 14 summarizes the representative pattern of medium-vessel vasculitis (reproduced from Saadoun, D., Vautier, M., Cacoub, P., Medium- and Large-Vessel Vasculitis, *Circulation*, 143, 3, by permission of Wolters Kluwer Health, Inc.).^38^ The dominant forms of medium-vessel vasculitis are polyarteritis nodosa and Kawasaki disease. This relationship is not mutually exclusive. These two vasculitides can manifest in other vessels as well, and the medium vessels can still be affected by the remaining vasculitides.

Supplementary Figure 14.

#### Polyarteritis nodosa

Polyarteritis nodosa (PAN) is a necrotizing vasculitis characterized by weight loss, asthenia, fever, peripheral neuropathy, hypertension, heart failure, gastrointestinal involvement, musculoskeletal involvement, renal involvement, or cutaneous lesions. Raynaud vascular issues occur in 20% of cases. In roughly 5% of patients, digital necrosis can be observed. PAN diagnosis, on paper, requires histological confirmation of segmental fibrinoid necrosis. Typically, the diagnosis can still be made based on angiographic findings, absence of antineutrophil cytoplasmic antibodies, and other pertinent clinical findings.^38^

MR angiography is an established method to examine the scope and extent of PAN and other large- and medium-vessel diseases. It allows observation of early inflammatory vasculitis pathology such as mural thickening. Enhancement of affected vessels can often be observed. Mural inflammation is best evaluated by *T*_1_ weighted, fat-suppressed, contrasted-enhanced sequences, and *T*_2_ weighted black-blood imaging. Common imaging findings include 1–5 mm aneurysms, often within hepatic, mesenteric, and renal branches. These can coincide with stenotic lesions due to fibrosis of adjacent vasculature. Newer sequences like 3D volumetric isotropic TSE acquisition can be combined with contrast-enhanced MRA for shorter acquisition time (compared to the 2D sequence) and assessment of stenosis and vessel wall changes. The main disadvantage of MRA in PAN is the considerable time required compared to CTA.^39^

#### Kawasaki disease

Kawasaki disease (KD) is a self-limited vasculitis that affects children under the age of 5. Coronary artery disease is present in about 20% of cases, which can lead to lethal or nonlethal infarction. KD typically presents with fever, bilateral non-exudative conjunctivitis, erythema of the oral mucosa and lips, rash, lymphadenopathy, and other inflammatory findings. The clinical diagnosis is made by at least 5 days of fever, and four of the five following criteria:

1. acute injury reactions such as desquamation of fingers and toes, peripheral edema (particularly in the distal extremities), erythema of the palm and soles;
2. polymorphous exanthema, diffuse maculopapular eruption, scarlatiniform erythredema;
3. bilateral bulbar conjunctival injection;
4. changes in lips and oral mucosa;
5. cervical lymphadenopathy.

If less than four of these categories are met, fever and confirmed coronary artery abnormalities can be detected.^38^ Multimodality imaging using MRA or CTA is recommended. MR imaging provides more information on the coronary anatomy as well as more information regarding ischemia, infarction, inflammation, and function, but CT is an effective alternative as well. Supplementary figures 15 and 16 demonstrate classical patterns of vessel involvement in Kawasaki disease, similar findings would be seen with CTA and MRA.^40,41^ The choice is largely based on availability and expertise.^39^

Supplementary Figure 15.

Supplementary Figure 16.

### Small vessel vasculitides

#### Lupus vasculitis

Lupus vasculitis (LV) is a secondary vasculitis due to systemic lupus erythematosus (SLE), most commonly associated with small vessels and occasionally medium-sized vessels. LV occurs in 50% of patients diagnosed with SLE and can involve different organ systems. Depending on the location and size of the vessels affected in LV, there is a large spectrum of clinical manifestations.^42^ Some life-threatening manifestations include mesenteric vasculitis, pulmonary hemorrhage, or mononeuritis multiplex. Therefore, early recognition and treatment are essential for improving patient outcomes.^43^

Most cases of lupus vasculitis commonly involve cutaneous manifestations; although visceral manifestation is less common, it causes more severe disease. LV also may occur in the central nervous system, peripheral nervous system, gastrointestinal system, kidneys, lungs and retina.^44^ Central nervous system vasculitis in SLE is commonly diagnosed through a combination of neuroimaging with clinical presentation; however, the gold-standard is a brain biopsy which is highly invasive and provides limited sensitivity.^45^ Therefore, MRI is the most sensitive non-invasive imaging technique for central nervous system vasculitis. A major downside of this technique is that it does not detect small-vessel vasculitis, even though it can capture wall-thickening and intramural contrast material uptake in large artery vasculitis.^46,47^ A more accurate diagnosis and differentiation between vasculopathies may be obtained by contrast-enhanced angiography since it can detect changes in vessel lumen, such as narrowing.^44^

In a 2015 case study, diagnosis of lupus-associated intestinal vasculitis was mainly dependent upon CT since the laboratory and clinical symptoms were non-specific. In lupus-associated intestinal vasculitis, CTA may reveal bowel wall thickening, bowel wall contrast enhancement, stenosis or engorgement of mesenteric vessels, and ascites, as illustrated by Supplementary figure 17a–b.^48^

Supplementary Figure 17.

#### Rheumatoid vasculitis

Rheumatoid vasculitis (RV) is the most serious extra-articular complication of rheumatoid arthritis (RA). It arises in patients with longstanding and severe RA, and it involves inflammation of small- and medium-sized vessels. It is also associated with a high morbidity rate due to organ damage from vasculitis and a high mortality rate with up to 40% of patients dying within 5 years.^49^ Although the incidence of RV has significantly decreased, the mortality rate remains high, which emphasizes the need for early diagnosis and effective treatment.^50^

RV typically presents with similar symptoms as other connective tissue diseases and infections, such as purpura, cutaneous ulceration, and gangrene of distal parts of the extremities. Therefore, it is necessary to exclude other diagnoses to confirm RV. RV is typically diagnosed based on a history of RA and histopathological evidence; however, there is no validated diagnostic criteria for RV. Laboratory tests are only supportive and provide no role in diagnosing RV, so skin biopsy is the least invasive and mostly used diagnostic tool.^51^ Imagining studies have no consistent role in the detection of RV, but angiography is occasionally used when gastrointestinal involvement is indicated. Angiography is appropriate when investigating mesenteric vasculitis or medium vessel vasculitis in the extremities.^51^ The minimal usage of CT or MR angiography in diagnosing RV may be due to the decreased prevalence of diagnosis in only less than 1% of patients with RA, as well as the primary usage of other diagnostic tools like histopathology.^52^

#### Sarcoid vasculitis

Sarcoid vasculitis (SV) is a rare complication of sarcoidosis that affects all vessel sizes and is most commonly associated with pulmonary sarcoidosis.^53^ Adults afflicted by SV demonstrated involvement of the abdominal aorta, pulmonary granulomatous angiitis, cutaneous vasculitis and granulomatous glomerulonephritis. Among children with sarcoidosis, a variety of vasculitis exists, including leukocytoclastic vasculitis, systemic vasculitis of small- to medium-sized vessels and large-vessel vasculitis.^54^

In one study by Fernandes et al,^55^ they found the presence of vasculitis in sarcoidosis was confirmed based on histologic findings of biopsy or by arteriographic features compatible with large vessel vasculitis. Arteriography showed medium- or large-vessel vasculitis demonstrated by stenosis, occlusion, or aneurysms, as seen in Supplementary figure 18.^55^ Clinical outcomes of patients with sarcoid vasculitis vary based on the vessels affected and treatment. Outcomes range from complete recovery after prednisone therapy to severe morbidity. Therefore, it is critical to implement vessel imaging, on top of histological findings, to guide interventions.

Supplementary Figure 18.

#### ANCA-associated vasculitis

A group of patients with small-vessel vasculitis, involving arterioles, capillaries, and venules, and glomerulonephritis have serum anti-neutrophil cytoplasmic antibodies (ANCA).^56^ ANCA are produced by T cells and activate leukocytes and monocytes, which result in damage to blood vessel walls, further attracting more leukocytes and prolonging the inflammatory process.^57^ ANCA antibodies are of two types, anti-proteinase-3 (PR3) or anti-myeloperoxidase (MPO). Granulomatosis with polyangiitis (GP), microscopic polyangiitis (MP), and eosinophilic granulomatosis with polyangiitis (EGP) belong to this group of vasculitides.^56,57^ Literature is sparce regarding the use of vessel imaging findings of this category of vasculitis, likely due to a low prevalence rate, and criteria for diagnosis is consistent with clinical findings, serum ANCA titers, and histopathology findings.^58^ Once diagnosed, as mortality is high without treatment, all patients receive urgent treatment with induction therapy involving immunosuppressants. Plasmapheresis is recommended in rapidly progressive renal disease or pulmonary hemorrhage.^59^

Granulomatosis with polyangiitis, formerly known as Wegener’s granulomatosis, is a disease that classically presents with fever, purulent rhinorrhea, nasal ulcers, sinus pain, polyarthralgias/arthritis, cough, hemoptysis, dyspnea, hematuria, and subnephrotic proteinuria; occasionally it may also present as cutaneous purpura and mononeuritis multiplex. Patients may present without renal involvement, although most will develop renal injury over the course of the disease. This disease is more common in patients with α_1_-antitrypsin deficiency, or those who have been exposed to silica dust.^60^ Chest X-ray is often remarkable for nodules and persistent infiltrates, sometimes with cavities. Biopsy of this tissue will reveal small-vessel vasculitis and adjacent non-caseating granulomas.^61^ Renal biopsy during active disease will depict segmental necrotizing glomerulonephritis without immune deposits and be characterized as focal, mixed, crescentic, or sclerotic.^62^ Relapse following remission is common and occurs more commonly than in patients with other ANCA-associated vasculitis, rendering necessary follow-up care.^56^

Microscopic polyangiitis presents like GP clinically, except these patients rarely have destructive sinusitis or significant lung disease, however, the distinction for diagnosis is made on biopsy, where granulomas are not present in MP. Further, similar to polyarteritis nodosa (PN), MP may lead to aneurysm formation. In the presence of glomerulonephritis, the most common manifestation of MP, kidney biopsy may demonstrate classic vascular changes in both PN and MP, however, there is risk of aneurysm rupture. There have been two documented cases of renal aneurysms that were diagnosed as MP using CTA following positive ANCA titers, allowing for the implementation of immunosuppressive therapy. Follow-up CTA after treatment was remarkable for the resolution of all previously observed abnormalities, demonstrating the effectiveness of CTA as a useful alternative to kidney biopsy in establishing the extent and progression of renal disease, in addition to response to treatment.^63^

Eosinophilic granulomatosis with polyangiitis (EGP), formerly known as Churg-Strauss disease, involves peripheral eosinophilia, cutaneous purpura, mononeuritis, asthma, and allergic rhinitis. Hyper-IgE and the presence of rheumatoid factor have been associated with the allergic state, and sole lung inflammation, involving cough and pulmonary infiltrates, often precedes systemic manifestations of the disease. Renal biopsy confers demonstrates focal segmental glomerulonephritis without immune deposits.^64^ EGP confers a worse prognosis and higher morbidity rate when involving the cardiovascular system, and cardiac MRI has been proven to be the most sensitive diagnostic technique consistently across many studies—outperforming the use of CTA.^65^ In a study by Dalia et al, one patient’s diagnosis was made via cardiac MRI in the setting of no stenosis, calcified plaque, or soft plaque on evaluation with CTA, demonstrating the greater accuracy and efficacy of cardiac MRI as the preferred diagnostic tool in EGP.^66^

#### Variable-vessel vasculitis

In variable-vessel vasculitis, there is no predominance of vessel size involvement. Behçet disease is a systemic variable-vessel vasculitis, most common in patients from the Middle East, Mediterranean, and Far East. The diagnosis is made clinically with the presence of oral ulcers and two of four: recurrent genital ulcers, skin lesions, eye lesions, and pathergy.^67^ Cogan’s syndrome is mitations to MRA assessmentcharacterized by ocular and audiovestibular symptoms, and approximately 70% of patients experience systemic symptoms.^68^

Often, tissue biopsy of certain large or medium vessels is not performed, necessitating imaging for diagnostic purposes. Available literature is limited regarding imaging features in Cogan and Behçet vasculitides, however, case reports and series have demonstrated their similarities with the large-vessel vasculitides. Spatial resolution further limits discussion on the variable small-vessel findings.^69^

#### Immune complex small vessel vasculitis

Overall, angiography plays a minor role in the diagnosis and management of immune complex small vasculitis. Nonetheless, a brief discussion of the immune complex small vasculitides follows.

#### IgA vasculitis

Also known as Henoch-Schönlein Purpura (HSP), this IgA immune complex deposition disease typically involves several organs, including the GI tract, skin, synovial membranes, and kidneys. It most commonly afflicts children between 4 and 7 years old, with clinical features including palpable purpura in dependent areas, arthralgias, arthritis, abdominal pain, melena, glomerulonephritis, and scrotal swelling.^70^ The role of angiography is unclear in the diagnosis and management of IgA Vasculitis. However, axial *T_2_* weighted MRI may show scattered hyperintense areas throughout the brain parenchyma, portrayed in Supplementary figure 19.^71^

Supplementary Figure 19.

Some case reports describe the utility of CTA in the diagnosis of HSP when clinical signs are not convincing. Specifically, the gastrointestinal symptoms of HSP may mimic a variety of other disorders, and imaging can be ordered to narrow an otherwise broad differential diagnosis. In one case, a 15-year-old male presented with skin rashes, joint pain, abdominal pain, and melena. A CTA of the abdomen was performed which demonstrated engorged mesenteric vessels, with multifocal areas of symmetric small bowel wall thickening with intervening normal segments. These non-specific vessel abnormalities were further corroborated by biopsy of skin rash demonstrating leukocytoclastic vasculitis. Thus, CTA alone was diagnostically insufficient, but provided further clues to narrow clinical decision-making. Another report of an 8-year-old male with acute onset skin rash and severe abdominal pain demonstrated similar findings on CTA as above. Mesenteric vessels were diffusely engorged. Other associated findings included symmetric thickening of bowel wall with mural stratification, with few subcentimetric mesenteric lymph nodes. Ultimately, both patients were treated with short courses of oral steroids and supportive care which resulted in a full recovery after seven days.^71^ Although these cases demonstrate the need for a comprehensive work-up, imaging studies were vital in the differentiation of vasculitis versus mesenteric ischemia. The presence of skip lesions with intervening normal segments, with involvement of unusual locations in the intestinal tract are more suggestive of an inflammatory vasculitis-associated process rather than acute ischemia due to embolism or thrombosis. Further, vasculitides are more likely to transcend the anatomic boundaries vascular fields.^71^

#### Anti-glomerular basement membrane disease

Anti-glomerular basement membrane (anti-GBM) antibody disease, or Goodpasture Syndrome, is an autoimmune disease involving inflammation and damage of alveolar and renal glomerular basement membranes by a cytotoxic antibody. The disorder typically presents as pulmonary hemorrhage and glomerulonephritis.^72^ Much like other small vessel vasculitides, CTA does not currently play a major role in the diagnosis and management of anti-GBM antibody disease. The manifestations of diffuse pulmonary hemorrhage from this vasculitis are readily apparent on CT, which will typically demonstrate lobular ground-glass attenuation and smooth interlobular and centrilobular interstitial thickening.^73^

Most cases of anti-GBM antibody disease will not need CT angiography for diagnosis. However, advanced angiographic imaging studies become valuable in clinically blurry cases. One case featuring a 17-year-old male who presented with a history of three hospital admissions over a two-month period for hemoptysis and shortness of breath was notable for a negative serological status for Goodpasture’s disease. However, on CTA of the lungs, florid and diffuse alveolar infiltrates. These imaging findings, in the context of the overall clinical picture were more suggestive of an autoimmune vasculitis than an infectious pneumonia. Although the patient was initially treated with antibiotics to no avail, the results of the CT pulmonary angiogram were enough to shift the clinician’s thought process. Subsequently, a repeat anti-GBM antibody test was ordered, which was positive. The diagnosis was confirmed on renal biopsy.^74^

#### Cryoglobulinemic vasculitis

Usually associated with other diseases, cryoglobulinemic vasculitis (CV) is an immune complex-mediated inflammation of blood vessels commonly seen in patients with underlying hepatitis C virus infection. Typically affecting small- and medium-sized blood vessels, CV causing inflammation through complement activation.^75^ Cryoglobulins are immunoglobulins that precipitate *in vitro* from plasma when temperatures fall below 37C.^76^ According to the Brouet classification, there are three major subgroups of cryoglobulins. Type 1 includes monoclonal immunoglobulins, typically IgM, which form in setting of plasma cell dyscrasias such as multiple myeloma and monoclonal gammopathy of unknown significance. Other causes of Type 1 cryoglobulinemia include Waldenström macroglobulinemia, non-Hodgkin lymphoma, and chronic lymphocytic leukemia.^77^ Type 2 consists of monoclonal IgM with rheumatoid factor activity and polyclonal IgG, and Type 3 involves polyclonal IgM with rheumatoid factor activity and polyclonal IgG.^78^

When these cold cryoglobulin immune deposits precipitate in the small vessel walls of extremities in cold temperatures, they cause leukocytoclastic vasculitis and palpable purpura.^79^ However, 4–8% of patients with CV may develop cardiac manifestations, including pericarditis, myocardial ischemia, or acute congestive heart failure.^80^ Evidence of coronary artery involvement is possible, typically resulting in focal areas of stenosis or aneurysm. These findings signify poor prognosis, with a median 2-year mortality rate of 50%.^81^ CTA and MRA can be used to detect these lesions, but ultimate diagnosis typically relies on serologic studies, specifically the cryoprecipitation test.

#### Hypocomplementemic urticarial vasculitis

Similar to CV, hypocomplementemic urticarial vasculitis (HUV) involves immune complex depositions leading to vasculitis which manifests as chronic urticaria with episodes occurring during low ambient temperatures, emotional stress, and even sometimes spontaneously. Low serum complement measurements in patients suggest that activation of the classical complement pathway is responsible for the symptoms. This activation leads to subsequently decreased C1q, C3 and C4. Classical clinical manifestations of HUV syndrome include recurrent episodes of chronic, nonpruritic, urticarial skin lesions with the potential for systemic involvement, *i.e.,* abdominal pain, respiratory disease, arthralgia and arthritis, glomerulonephritis, and leukocytoclastic vasculitis. Over of half of patients can be present with angioedema, often involving the facial area and upper extremities.^82,83^ Although angioedema most commonly involves the face and upper extremities, other rarer presentations of angioedema may prove to be a difficult diagnosis.

Coulier et al describe two cases of extensive cervico-thoraco-abdominal attacks of angioedema. In both cases, the only external sign of edematous infiltration was swelling in the left supraclavicular fossa. In the first case, contrast-enhanced thoracoabdominal CT demonstrated massive, continuous edema involving deep tissues extending from the neck to the pelvis. Areas typically unaffected by angioedema such as the mediastinum and axial retroperitoneum were heavily implicated in these angioedema attacks. Supplementary figure 20a–d demonstrates the massive cervical edema, mediastinal edema, and diffuse retroperitoneal edema, as indicated by the black star, black arrow, and white arrows, respectively.^84^ Supplementary figure 21a–d demonstrates these same findings using the corresponding markers as described for Supplementary figure 20a–d.^84^ Note the lack of other signs of third spacing, specifically the lack of any radiological findings suggestive of pleural or pericardial effusion, or ascites. Further, lack of colonic wall thickening suggests no edematous infiltration of the gastrointestinal tract.^84^ Although the most common causes of angioedema stem from bradykinin or histamine-mediated responses, vasculitis is another potential etiology. HUV syndrome remains the most common vasculitis associated with angioedema. However, PAN involving the splanchnic arteries supplying the gastrointestinal tract may be responsible for bowel wall thickening due to edematous infiltration.

Supplementary Figure 20.

Supplementary Figure 21.

Some precipitates were identified as C1q, thus, the disease is occasionally referred to as anti-c1q vasculitis.^85^ Diagnosis requires biopsy and serum tests, however, there are some suggestive imaging findings.^36^ Cardiac MRA may demonstrate irregularities in the coronary vascular walls, as well as patchy areas of enhancement in the mesocardium. These findings of mild vascular involvement with skip lesions and resultant abnormalities in the myocardium indicate a likely inflammatory vasculitis with secondary myocardial damage.^36^

An even more severe, but rare, complication of HUVS is cardiac valvular involvement. Extensive literature review revealed only 10 total cases of HUVS with cardiac valvular involvement.^12,86–93^ The presence of abnormalities of the cardiac valves in the setting of HVUS represents a poor prognostic factor. Although rare, the typical cardiac involvement of HVUS is characterized by acute necrotizing endocarditis and fibrin deposition on the surface of valve leaflets leading to fibrocalcific degenerative changes. Since these changes are nonspecific on cardiac imaging, the main role of the radiologist’s interpretation is in prognostication in the setting of an established HVUS diagnosis.^86^

## Conclusion

Increased availability and higher resolution of modern imaging techniques have allowed clinicians to diagnose rare vasculitides with greater precision and accuracy than ever before. Although most of these diseases remain a clinical diagnosis, recommendations and guidelines for the use of imaging in the diagnosis of vasculitis is ever increasing. Pathognomonic signs of large vessel vasculitides have been established. However, there is far more uncertainty in the smaller vessel vasculitides, and more clinical research in the use of imaging in these disorders will pave the way to elucidating their manifestations on imaging. As imaging finds wider use in the management of vasculitides, different disease phenotypes may be uncovered. Through objective analysis, the often-blurred margins that separate the different small vessel vasculitides may become concrete radiologic diagnoses. Through collaboration between rheumatology and radiology, the diagnosis and management of all vasculitides will be revolutionized. Lastly, it is important to perform imaging as soon as possible. Many of the imaging abnormalities may vanish upon treatment initiation.
