# Supplementary figures and images for: MR and CT angiography in the diagnosis of vasculitides
Source: BJR Open. 2023 Sep 25;5(1):20220020. doi: 10.1259/bjro.20220020 (PMC10636354; doi:10.1259/bjro.20220020)

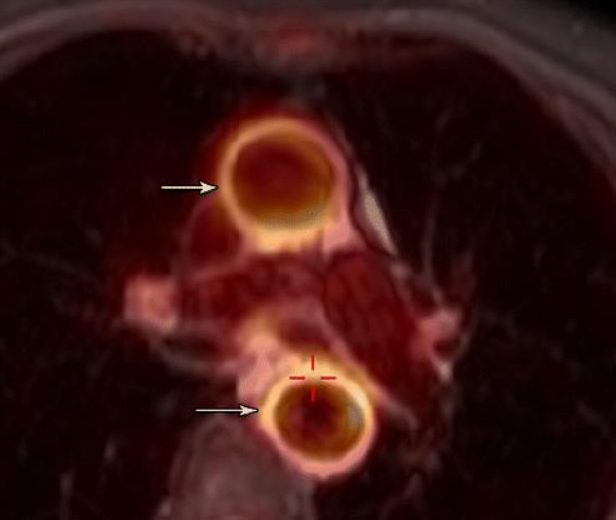

Supplement: Supplementary file 1 — Supplementary Figure 13. [file bjro.20220020.suppl-01.png]

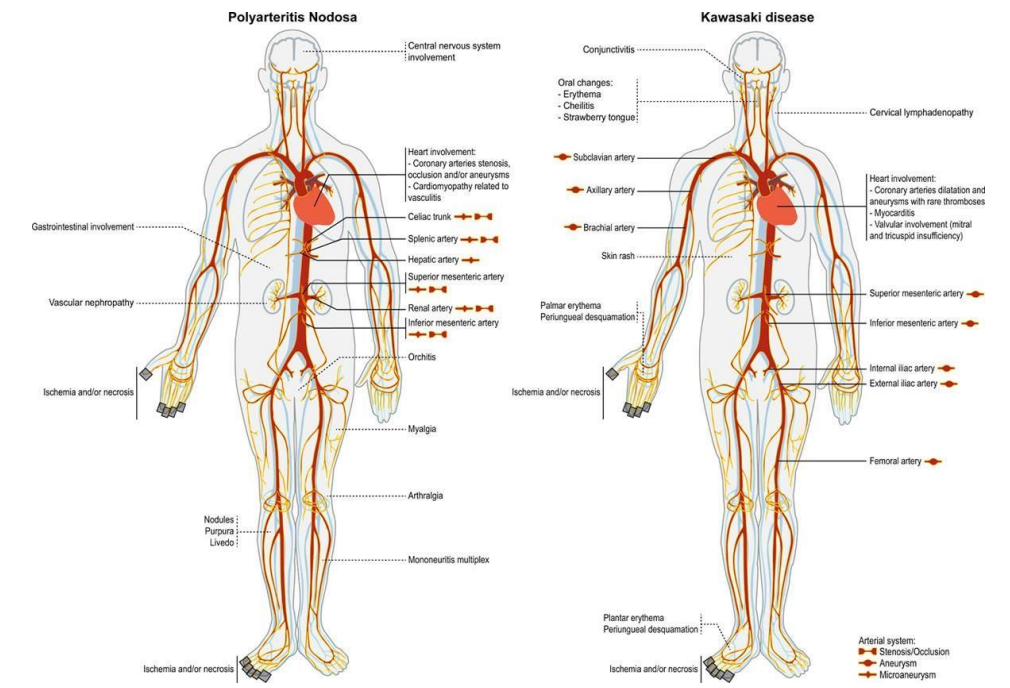

Supplement: Supplementary file 2 — Supplementary Figure 14. [file bjro.20220020.suppl-02.png]

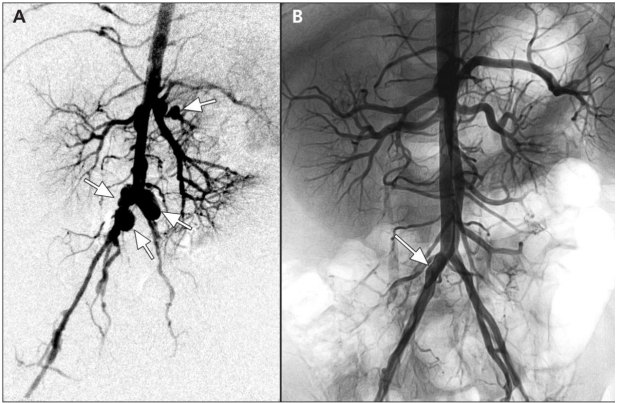

Supplement: Supplementary file 3 — Supplementary Figure 15. [file bjro.20220020.suppl-03.png]

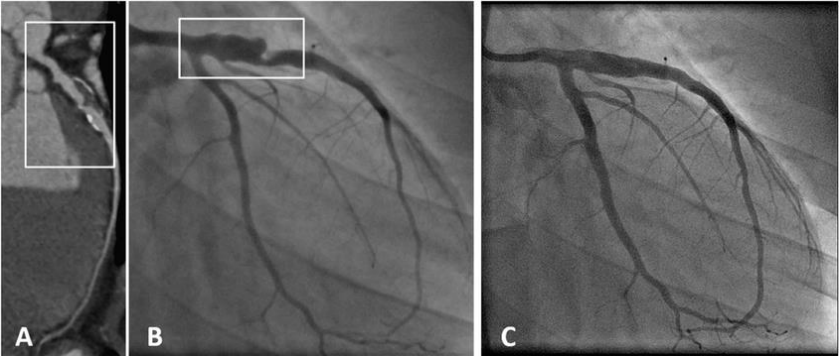

Supplement: Supplementary file 4 — Supplementary Figure 16. [file bjro.20220020.suppl-04.png]

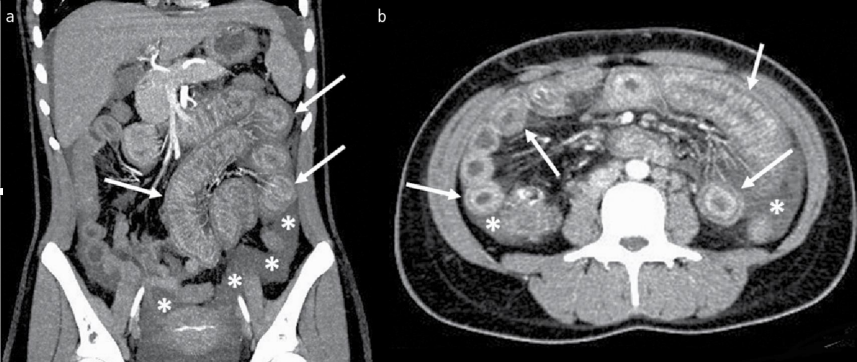

Supplement: Supplementary file 5 — Supplementary Figure 17. [file bjro.20220020.suppl-05.png]

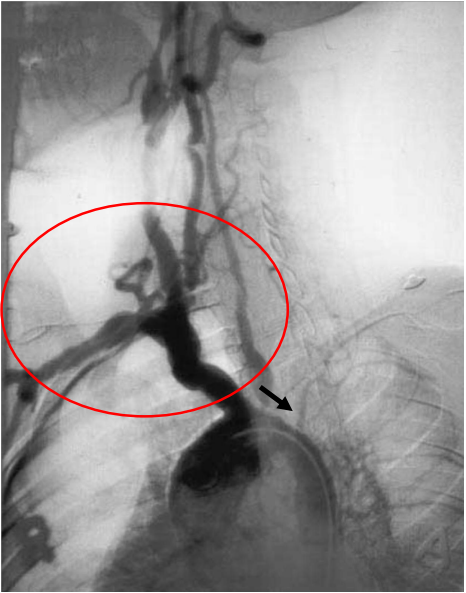

Supplement: Supplementary file 6 — Supplementary Figure 18. [file bjro.20220020.suppl-06.png]

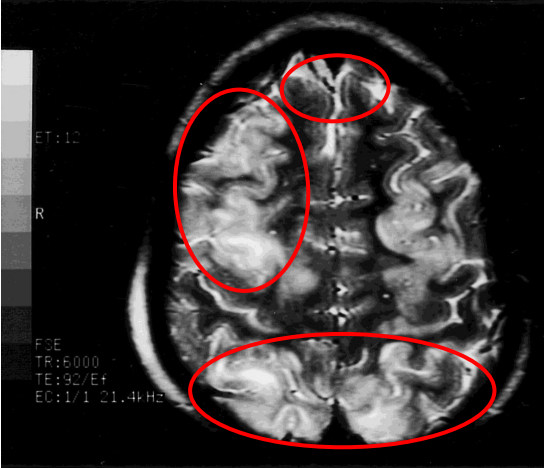

Supplement: Supplementary file 7 — Supplementary Figure 19. [file bjro.20220020.suppl-07.png]

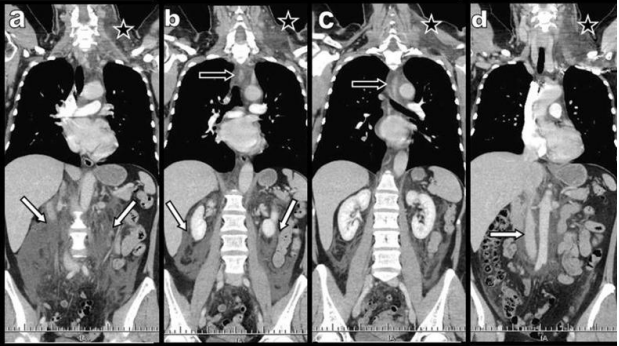

Supplement: Supplementary file 8 — Supplementary Figure 20. [file bjro.20220020.suppl-08.png]
